# Multimorbidity, Trauma Exposure, and Frailty of Older Adults in the Community

**DOI:** 10.3389/fgene.2021.634742

**Published:** 2021-03-24

**Authors:** Ioanna V. Papathanasiou, Evangelos C. Fradelos, Dimitrios Mantzaris, Anna Rammogianni, Foteini Malli, Dimitrios Papagiannis, Konstantinos I. Gourgoulianis

**Affiliations:** ^1^Community Nursing Lab, Faculty of Nursing, University of Thessaly, Volos, Greece; ^2^General Department, University of Thessaly, Volos, Greece; ^3^Computational Intelligence and Health Informatics Lab, Faculty of Nursing, University of Thessaly, Volos, Greece; ^4^National Organization of Public Health, Refugees Reception Centre of Agia Varvara, Veroia, Greece; ^5^Faculty of Nursing, University of Thessaly, Volos, Greece; ^6^Respiratory Medicine Department, Faculty of Medicine, University of Thessaly, Volos, Greece

**Keywords:** multimorbidity, chronic diseases, trauma exposure, frailty, older adults, community health, health care

## Abstract

The aim of this study is to investigate the relation between multimorbidity, traumatic events and frailty among older adults in the community. The studied population consisted of 257 older people who were recipients of the services and active members of Open Care Centers for the Elderly (OCCE) of the Municipality of Grevena and meet a set of selection criteria. The collection of the data was carried out using a fully structured questionnaire, which consisted of two sections: a form of individual features and the Tilburg Frailty Indicator (TFI). The sample consisted of 114 men (44.4%) and 143 women (55.6%) aged between 61 and 96 years with an average of 75.12 years. The results showed that the mean scores were 2.70 for the Physical Frailty (standard deviation = 2.16), 1.43 for the Psychological Frailty (standard deviation = 1.21), 1.32 for the Social Frailty (standard deviation = 0.64) and 5.44 for the total Frailty (standard deviation = 3.02). We took into account the cut-off point five of 54.1% (*n* = 139) in terms of the participants’ frailty. Physical, Psychological, and Total Frailty are related to (a) the presence of two or more chronic diseases or disorders, (b) the experience of a serious illness in the previous year, and (c) the experience of a serious illness of a loved one during the previous year. The outcomes helped to identify frailty syndrome in older people and the factors associated with it.

## Introduction

European countries are among the countries in the world with the highest population of aging people according to epidemiological data (Kinsella and Philips, 2005). The demography of Greece shows a simultaneous and constant increase in people who are aging and those with chronic diseases (Ntanasi et al., 2018). Greece is second in Europe in aging population after Italy (Sintihaki and Kasabali, 2010). Data from recent literature show that a common phenomenon observed in older adults and especially in those suffering from chronic diseases is the frailty syndrome, which affects not only their physical and mental health but also their social life (Vardaki and Manolitsaki, 2011; Koutsonida et al., 2016).

The frailty syndrome has been defined several times by researchers; however, no common definition has prevailed worldwide (Chen et al., 2014). From Fried et al. (2001), frailty has been defined as a biological syndrome that has detrimental effects on the body’s systems due to limited resistance to stressor factors. Other scientists declare that it is a clinical syndrome affecting older people, as it can cause a decrease in functionality, a rise in the probability of falls, and possibly an increase in hospitalization, disabilities, and mortality (Xue, 2011). In the study by Conroy and Elliott (2017), frailty is a multidimensional geriatric syndrome with significant health effects.

The frailty syndrome is featured by five phenotypic criteria, according to Fried et al. (2001), while the occurrence of one or two of them can be characterized as a predisposition syndrome. These criteria include energy loss, decreased physical activity, decreased gait, weakness, and weight loss. The frailty existence is due to biological, genetic, physical, environmental, social, and psychological factors. This reasoning results from the five criteria of Fried et al. (2001). The featured conclusion of the research was that females are prone to frailty syndrome, while other factors that have been associated with the occurrence of the syndrome are low socioeconomic status, education level, lifestyle (alcohol use and smoking), the psychological situation, such as the presence of depressive symptoms, and disability (Rockwood et al., 2004; Theou et al., 2014; Lewis et al., 2019).

Several multidimensional instruments are currently available for measuring frailty in older persons, such as the Tilburg Frailty Indicator (TFI), the Edmonton Frail Scale, the Frailty Index and the Groningen Frailty Indicator. The TFI differs from these instruments in that the score on the TFI results entirely from self-reports and contains no questions on disability. Research has shown that frailty should be distinguished from disability as it is regarded as a pre-disability state (Gobbens et al., 2012).

The syndrome and therefore the modern diseases are caused by way of life and stress despite the continued improvement of hygiene and nutrition. Frailty is associated with chronic diseases, including cognitive impairment, as several studies have remarked that frail older adults were highly affected by some type of dementia (Kulmala et al., 2014; Rogers et al., 2017; Lewis et al., 2019).

Fried et al. (2001) published one of the most valuable papers on the frailty syndrome, researching the prevalence, frequency, accuracy, and prediction of the clinical syndrome. This was a large-scale study that was completed in 7 years. The researchers observing demographic characteristics (lifestyle), health habits, medication intake, weight loss, the presence of cardiovascular events, asthma, diabetes, visual, and hearing impairment in the older people, conducted an assessment of physical activity, mental state, cognitive function as well as morbidity and mortality. After 5 years, in the observed population, the percentage of frailty doubled in women. Moreover, there was an observed 7% increase in frailty after 3 years in people over 90 years old. In frail older people, 27% did not have any kind of disability or comorbidity. The cohort study shows that individuals with the following features are more prone to frailty: low income, comorbidity, chronic illness, and a low educational level (Fried et al., 2001). Frailty can also cause disability and can be associated with obesity (Fried et al., 2001).

Frailty and multimorbidity are considered promising clinical biomarkers for studying mechanisms underlying aging. Both have been shown to be related to the risk of disability in older people, hospitalization, mortality, and escalating health-related costs. A certain degree of overlap between the two conditions is biologically plausible, and bidirectional causality between them is likely. Frailty may predispose individuals to the development of multiple chronic diseases, but it may also stem from the coexistence of multiple conditions (Vetrano et al., 2019). Evidence suggests that frail older people are a clinically highly heterogeneous group and that further risk stratification may allow the identification of subgroups at greater risk. Also, the accumulation of chronic health problems, along with age-related physiologic changes, results in multisystem dysregulation, leading to frailty. Different chronic disease profiles, or patterns of multimorbidity, which is defined as the co-occurrence of two or more chronic conditions, may impersonate clinically different etiologic pathways to frailty and provide additional prognostic information within older adults with the same level of frailty (Nguyen et al., 2019).

Traumatic life events have been associated with having long-term effects on human health and causing early late-life mortality, and it can thus be argued that frailty must be associated with traumatic life events. In the conceptual model of frailty proposed by Freitag and Schmidt (2016). traumatic life events are described as part of sociodemographic factors affecting the development of diseases and frailty. Despite the empirical findings on health and theoretical links with frailty, the association between traumatic life events and frailty has not been investigated in much detail so far (Freitag and Schmidt, 2016).

This study aims to address this research gap and investigate the relationship between multimorbidity, traumatic events and frailty among older adults in the community.

## Materials and Methods

### Research Methodology and Sample

A descriptive epidemiological, cross-sectional study was performed to be considered the correlation between multimorbidity, traumatic events and frailty among older adults in the community. The studied population consisted of the older adults who were recipients of the services and active members of the Open Care Centers for the Elderly (OCCE) of the Municipality of Grevena. We recruited participants for this study by visiting the three OCCE of the Municipality of Grevena, one urban structure and two country centers of Grevena. A sample of 257 older people was collected from the interested population. Individuals participated in this research voluntarily, and all of them provided written informed consent.

The selection criteria of the individuals consisting the study population were as follows:

a. People greater than 60 years old.
b. Active members of (OCCE) of the Municipality of Grevena.
c. Self-service and independent living.
d. Undiagnosed mental or cognitive disorder.
e. Knowledge of the Greek language and fluency of communication.
f. Acceptance of the terms and participation in the research.

### Data Collection

The applied sampling method was the non-probability sampling and, in particular, the convenience sampling technique. The collection of the research data by the research team took place in the three OCCE of the Municipality of Grevena following the acquisition of a relevant permit and the approval of the research by the competent service of the Municipality. The questionnaires were completed by the older adults themselves after being informed about the purpose of the research, and the required clarifications were given. The participation of the older people was voluntary and in accordance with the principles and ethical rules of the research.

### Questionnaire

The collection of the empirical research material was carried out using a special and fully structured questionnaire, which consisted of the following two sections.

#### Sociodemographic Variables, Living Status and Health Assessment Questionnaire

The questionnaire is specifically designed for the present study and includes some self-reported questions taken from part A of TFI without any changes and some questions from part A of TFI adjusted to the sociocultural context of Greece; we also added some other questions. In particular, the questionnaire includes questions about the socio-demographic information of older people (gender, age, marital status, number of children, educational level, permanent residence area and individual monthly income) as well as the information mentioned in the self-assessment of health and living status (living alone or with other persons, healthy lifestyle, the existence of chronic diseases or disorders, the experience of psychologically stressful situations and satisfaction from the family environment).

#### Tilburg Frailty Indicator

The TFI was used to assess the frailty of older people (Gobbens et al., 2010). The TFI was chosen as it is a self-administered instrument for screening for frailty in older adults and also it is an instrument that is economical, efficient and quick to provide answers, which has proven to be reliable and valid (Freitag et al., 2016). The TFI Frailty Scale has satisfactory psychometric characteristics of reliability and validity when tested on samples of older people living in the community. Zhang et al. (2020) examined the internal consistency, convergent and divergent validity, and concurrent validity of the TFI within a diverse community-based sample of older adults in five European countries, including Greece. The Cronbach’s alpha of the physical domain in three countries, including Greece, varied between 0.60 and 0.67 while the internal consistency of the psychological and social domains was not satisfactory in none of the studied countries, with the Cronbach’s alpha varying between 0.22 and 0.55. Also, regarding the full TFI, it had satisfactory reliability with an internal consistency of Cronbach’s α ≥ 0.70 in the total population and in each country. The authors concluded that the TFI was a reliable and valid psychometric instrument for use in screening for frailty in community-dwelling older people in Spain, Greece, Croatia, Netherlands, and United Kingdom (Zhang et al., 2020). The TFI Frailty Scale is a self-report questionnaire for the detection of frailty in older adults and consists of 15 questions that assess frailty in three sub-scales: “Physical Frailty,” which includes eight questions; “Psychological Frailty,” which includes four questions and “Social Frailty,” which includes three questions. A total of 11 questions accept binary answers (Yes or No), and the remaining four questions provide three options (Yes, Sometimes, and No); they are graded with values of zero or one. The total score of the questionnaire and the sub-scales is the sum of the answers to the individual questions that make them up. Higher score values declare higher levels of frailty in the respondent.

### Statistical Analysis

The processing and the statistical analysis of the empirical research material were done using the software package “SPSS 25.0 for Windows,” with the methods of Descriptive and Inferential Statistics. In particular, the Descriptive analysis included the frequency distribution for the qualitative variables (absolute and relative% frequency) as well as estimates of position and spreading parameters for the quantitative variables (mean, median, standard deviation, minimum and maximum value). The Kolmogorov–Smirnov test was used for assessing the normality of the distribution, which was *p* > 0.05, and a *t*-test was thus used to exam possible differences of frailty among groups of multimorbidity and traumatic events exposure. The significance levels (*p* value) were two-sided, and the level of acceptable statistical significance was set at *p* < 5%.

## Results

### Sample Characteristics

The sample of 257 older people consisted of 114 men (44.4%) and 143 women (55.6%). Their age ranged from 61 to 96 years with an average value of 75.12 years. Of these, 65.4% were married and 30.4% were widowed. Three out of five older people had one to two children (60.7%). Most of the older people were primary school graduates (68.5%) and lived in a non-urban area (54.9%). Regarding their monthly personal income, 55.6% of the studied population received between 500 and 1,000 euros. Approximately 3/4 of the older people lived with family members or others (75.9%). According the older people’s answers, 61.9% described their lifestyle as healthy, and 54.9% did not have two or more chronic diseases or disorders. In terms of experiencing stressful psychological experiences, 27.2% had experienced the death of a loved person, 19.1% a serious illness, 23.0% a serious illness of a loved one, 7.0% a divorce or the end of an important relationship, 0.8% a car accident and 2.3% a crime. Finally, the vast majority of older people (93.0%) reported that they were satisfied with the family environment. The Table 1 summarizes the aforementioned findings.

**TABLE 1:** Individual features of the older adults (*n* = 257).

Characteristics	*n*	%
**Gender**		
Male	114	44.4
Female	143	55.6
**Age (years)**		
Mean ± SD		75.12 ± 8.39
Min–Max		61–96
**Marital status**		
Single	4	1.6
Married	168	65.4
Divorced	7	2.7
Widowed	78	30.4
**Number of children**		
0	8	3.1
1–2	156	60.7
≥3	93	36.2
**Education level**		
Primary education	176	68.5
Secondary education	61	23.7
Tertiary education	20	7.8
**Permanent residence area**		
Urban	116	45.1
Non-urban	141	54.9
**Individual monthly income (Euro)**		
<500	63	24.5
500–1,000	143	55.6
>1,000	51	19.8
**Cohabitation**		
Alone	62	24.1
Family members or others	195	75.9
**How would you describe your lifestyle?**		
Healthy	159	61.9
Neither healthy nor unhealthy	90	35.0
Unhealthy	8	3.1
**Do you have two or more chronic diseases or disorders?**		
Yes	116	45.1
No	141	54.9
**Did you experience any of the following during the previous year?**		
Death of a loved one	70	27.2
Serious disease	49	19.1
Serious illness of a loved one	59	23.0
Divorce or the end of an important relationship	18	7.0
Car accident	2	0.8
Criminal action against you	6	2.3
**Are you satisfied with your family environment?**		
Yes	239	93.0
No	18	7.0

### Frailty

The internal consistency reliability of the Frailty Scale (TFI), determined by the Cronbach’s Alpha coefficient, was for the total Frailty α = 0.75, while in the three sub-scales it was α = 0.74 for the Physical Frailty, *a* = 0.68 for Psychological Frailty and α = 0.21 for Social Frailty. Consequently, the Social Frailty reliability was low, while the other dimensions of the TFI Scale showed very good reliability of internal consistency (Table 2).

**TABLE 2:** Frailty levels of the older adults.

Tilburg frailty indicator (TFI)	Cronbach’s alpha	Mean ± SD	Median	Min–Max values
Physical frailty	0.74	2.70 ± 2.16	2.00	0–8
Psychological frailty	0.68	1.43 ± 1.21	1.00	0–4
Social frailty	0.21	1.32 ± 0.64	1.00	0–3
Total frailty	0.75	5.44 ± 3.02	5.00	0–13

The score for the total Frailty ranged from zero to 13 with an average value of 5.44 (standard deviation = 3.02) and a median value of 5.00. The findings of the study showed that the majority of older people had relatively low values of overall Frailty, as the total scores of the 50% of the patients were below 5.00 (median), which is less than 7.5—the middle point of the theoretical range of responses (Table 2). Taking into account cut-off point 5, around 54.1% (*n* = 139) of the participants displayed frailty.

A study of the individual dimensional outcomes of the TFI Scale shows that the Physical Frailty score ranged between zero and eight with an average value of 2.70 (standard deviation = 2.16), the Psychological Frailty score ranged from zero to four with an average value of 1.43 (standard deviation = 1.21) and the Social Frailty score was between zero to three with an average value of 1.32 (standard deviation = 0.64) (Table 2).

### Chronic Diseases, Trauma Exposure and Frailty

The presence of two or more chronic diseases or disorders is related to the Physical (*p* < 0.001) and Psychological Frailty (*p* < 0.001) as well as to the Total Frailty (*p* < 0.001) of older people. In particular, the older people with two or more chronic illnesses or disorders have a higher mean value of Frailty levels than the older persons with one or no chronic illness or disorder (Table 3).

**TABLE 3:** Differences of frailty (TFI) among groups of chronic diseases and traumatic events exposure.

Features	TFI Scale			
	Physical Frailty	Psychological Frailty	Social Frailty	Total Frailty
**More than 2 chronic disease or disorders**				
Yes	3.56 ± 2.22	1.72 ± 1.21	1.29 ± 0.61	6.58 ± 2.92
No	1.99 ± 1.83	1.18 ± 1.16	1.33 ± 0.66	4.51 ± 2.78
*T*	6.098	3.642	0.504	5.802
*p* value	**<0.001**	**<0.001**	0.615	**<0.001**
**The experience of death of a loved person**				
Yes	2.97 ± 2.15	1.50 ± 1.15	1.36 ± 0.68	5.83 ± 2.95
No	2.60 ± 2.16	1.40 ± 1.23	1.30 ± 0.62	5.30 ± 3.04
*T*	1.234	0.583	0.647	1.253
*p* value	0.218	0.561	0.518	0.211
**The experience of a serious illness**				
Yes	3.55 ± 2.26	1.90 ± 1.26	1.35 ± 0.63	6.80 ± 3.19
No	2.50 ± 2.09	1.32 ± 1.17	1.31 ± 0.64	5.13 ± 2.89
*T*	3.122	3.071	0.388	3.566
*p* value	**0.002**	**0.002**	0.698	**<0.001**
**The experience of a serious illness of a loved person**				
Yes	3.47 ± 2.23	1.76 ± 1.17	1.29 ± 0.53	6.53 ± 2.89
No	2.47 ± 2.08	1.33 ± 1.21	1.32 ± 0.67	5.12 ± 2.99
*T*	3.199	2.444	0.372	3.193
*p* value	**0.002**	**0.015**	0.711	**0.002**
**The experience of a divorce or the end of an important relationship**				
Yes	2.61 ± 1.91	1.33 ± 0.97	1.50 ± 0.71	5.44 ± 2.64
No	2.71 ± 2.18	1.44 ± 1.23	1.30 ± 0.63	5.44 ± 3.05
*T*	0.182	0.344	1.281	0.001
*p* value	0.856	0.731	0.201	0.999

*Bold is used to emphasize the statistically significant results.*

The experience of a serious illness in the previous year is related to the Physical (*p* = 0.002) and Psychological Frailty (*p* = 0.002) but also to the Total Frailty (*p* < 0.001) of older people. Particularly, older people who experienced a serious illness in the last year have a higher mean value of Frailty than older adults who had no such experience (Table 3).

The experience of a serious illness of a loved one during the previous year is related to Physical (*p* = 0.002) and Psychological Frailty (*p* = 0.015) but also to the Total Frailty (*p* = 0.002) of older people. Specifically, older persons who experienced a serious illness of a loved one in the last year have a higher average value of Frailty levels than older people who did not live such an experience (Table 3).

## Discussion

The Greek population is aging rapidly and especially in the provinces. The aging of the population is a global fact that leads scientists to further engage and conduct research to address issues related to an older demographic. The purpose of this paper was to study the relationship between chronic diseases, traumatic experiences and frailty in older people living in the community.

The results of the present study presented that the existence of two or more chronic diseases or disorders is related to physical, psychological and overall frailty in the older people living in the community. Particularly, comorbidity is associated with higher frailty rates. A similar result was obtained by the study of Vergara et al. (2019) in which 865 people over the age of 70 participated, and they presented that comorbidity is associated with high frailty. The most common chronic diseases of older people, in the Vergara et al. (2019) study, were diabetes mellitus, heart failure and chronic obstructive pulmonary disease. The meta-analysis of 30 studies conducted by Skela-Savic and Gabrovec (2018) on the prevention of frailty syndrome and its management at the individual level, showed that comorbidity, being female and age were the most important factors related to the occurrence and prognosis of the syndrome.

The experience of traumatic events in the last year shows a positive statistically significant relationship with frailty. Specifically, older persons who in the previous year either experienced a serious illness themselves or experienced a serious illness of a loved one collected a higher average value of frailty levels than older people who did not experience such a traumatic event. Aguayo et al. (2018) in a study of 5,294 older people, researched the correlation between experiencing a previous traumatic event or serious illness and frailty. The study showed that loss, diseases such as cancer and cardiovascular disease and lifestyle are positively correlated with the frailty score (Aguayo et al., 2018). The relationship between frailty, disability, and the experience of a significant illness was also studied by Greco et al. (2014) who found that chronic kidney disease in aging is significantly associated with physical, psychological, and social frailty, which is mainly due to depressive symptoms and cognitive deficits. Freitag and Schmidt (2016) found that resilience is significantly associated with frailty, as participants in their study with high levels of quality of life and resilience were less likely to be frail. Another study found that frail adults have greater difficulty adjusting to and recovering from stressful situations and that, compared with non-frail adults, frail adults are maybe more likely to use less suitable strategies or to have less resources to deal with the perceived stress (Desrichard et al., 2018).

This study presents limitations, which makes it difficult to draw general conclusions; these include limitations such as the small sample and the sampling method. The percentage population of older people in the Municipality of Grevena is not great, and so the sample of the study includes only 257 people, and convenience sampling was performed. Many older persons refused to participate in the study, and many of them had difficulty completing the questionnaire due to health problems or a low level of education. The limitations of the present research imply the design and implementation of new studies in a larger sample, by selecting the members of the sample by random sampling, for greater validity of the results and their optimal verification. This will contribute to drawing valid conclusions so that, in the future, the implemented plans could improve the quality of care provided to older people.

However, despite the above limitations and considering that the results of the present study are in accordance with the outcomes of similar international studies. The findings are considered, by researchers, to be reliable and safe, both overall and in the individual parameters, and they are suitable for use in Greece as well as internationally, contributing to the appropriate and effective assessment of frailty and its relationship with comorbidity and the experience of traumatic events.

Moreover, further investigation is needed regarding the correlation between multimorbidity, traumatic events and frailty among older adults in the community, and, also, it would be useful to investigate the types of strategies frail and non-frail adults use to cope with stressors (Desrichard et al., 2018). Research evidence on frailty should be translated into clinical practice and health care policymaking to improve quality care and promote healthy aging. This would also reduce the impact of aging on health care systems and strengthen their sustainability. Among others, the current challenges related to frailty research include the further understanding of interventions to reverse frailty and the best timing for intervention as well (Kojima et al., 2019).

## Conclusion

In conclusion, the study showed that the older people who participated in the research were not very frail. Nevertheless, the comorbidity and the experience of a serious illness, in the past, of both the older people and a loved one are important factors that are statistically and significantly associated with higher levels of frailty. The results of the study helped to identify the frailty syndrome in older people as well as the factors associated with it. However, conducting new studies on a larger sample in the Greek region will derive more reliable and accurate results. In addition, the necessity to provide new data to healthcare professionals will lead to their faster information, awareness, and education and training regarding the geriatric patients, and this will contribute to the optimization of healthcare.

## Data Availability Statement

The raw data supporting the conclusions of this article will be made available by the authors, without undue reservation.

## Ethics Statement

The studies involving human participants were reviewed and approved by the Ethics Committee of the Center of Social Solidarity and Sports of the Municipality of Grevena. The patients/participants provided their written informed consent to participate in this study.

## Author Contributions

IP, EF, and KG: conceptualization. DP: methodology and data curation. DM: software and formal analysis. IP, AR, and DM: investigation. AR: resources. IP and AR: writing—original draft preparation. IP, EF, DM, and KG: writing—review and editing. IP and EF: visualization. IP and KG: supervision. All authors read and approved the final manuscript and agree to be personally accountable for the authors’ own contributions and for ensuring that questions related to the accuracy or integrity of any part of the work**—**even ones in which the authors were not personally involved**—**were appropriately investigated.

## Conflict of Interest

The authors declare that the research was conducted in the absence of any commercial or financial relationships that could be construed as a potential conflict of interest.
